# 

**Published:** 1890-05

**Authors:** 


### A CHANGE FOR THE BETTER.

Our patrons must have observed the improved appearance of our Journal, commencing with the April issue. That the change was needful, and has been thorough, a comparison of the April and May numbers with previous ones, will sufficiently illustrate.

We shall spare neither pains nor money to furnish our patrons with a HEALTH monthly, second to no other of its class. All we ask in return is that some of them will be a little more prompt in their remittances, for they are necessary to our work.

### LITERARY.

We are in receipt of Vol. I, No. 1, of the REVUE INTERNATIONALE DE BIBLIOGRAPHIE MÉDICALE, PHARMACEUTIQUE ET VÉTÉRINAIRE, Paris, France, and Beyrouth, Syria, 284 pp. The design of this work is to give in two yearly volumes, a brief review of all the known medical and surgical publications, the world over. The present volume is indicative of completeness in this new field.

ALSO,

Report of Yale University Department of Medicine, 1890.

Twenty-second Annual Report of the New York Orthopædic Dispensary and Hospital.

Rectal Diseases: Observations and Experiences; by E. F. Hoyt, M. D.

